# Comprehensive assessment of multiple tryptophan metabolites as potential biomarkers for immune checkpoint inhibitors in patients with non-small cell lung cancer

**DOI:** 10.1007/s12094-020-02421-8

**Published:** 2020-06-12

**Authors:** M. Karayama, J. Masuda, K. Mori, H. Yasui, H. Hozumi, Y. Suzuki, K. Furuhashi, T. Fujisawa, N. Enomoto, Y. Nakamura, N. Inui, T. Suda, M. Maekawa, H. Sugimura, A. Takada

**Affiliations:** 1 Second Division, Department of Internal Medicine, Hamamatsu University School of Medicine, 1-20-1 Handayama, Hamamatsu, 431-3192 Japan; 2 Global Application Development Center, Shimadzu Corporation, 3801 Hadano, Kanagawa, 259-1034 Japan; 3 Department of Respiratory Medicine, Shizuoka City Shimizu Hospital, 1231 Miyakami, Shizuoka, 424-8636 Japan; 4 Department of Laboratory Medicine, Hamamatsu University School of Medicine, 1-20-1 Handayama, Hamamatsu, 431-3192 Japan; 5 Department of Tumor Pathology, Hamamatsu University School of Medicine, 1-20-1 Handayama, Hamamatsu, 431-3192 Japan; 6 International Projects On Food and Health, Tokyo, Japan

**Keywords:** Anti-programmed death-1 therapy, Anti-PD-1 therapy, ICI, IDO, Immune therapy, Indoleamine-2,3-dioxygenase

## Abstract

**Purpose:**

Tryptophan metabolites have immunomodulatory functions, suggesting possible roles in cancer immunity.

**Methods:**

Plasma tryptophan metabolites were measured using liquid chromatography/mass spectrometry before immune checkpoint inhibitors (ICIs) in patients with non-small cell lung cancer (NSCLC).

**Results:**

The 19 patients with NSCLC had significantly lower levels of tryptophan (*p* = 0.002) and xanthurenic acid (*p* = 0.032), and a significantly higher level of 3-hydroxyanthranilic acid (3-HAA) (*p* = 0.028) compared with the 10 healthy volunteers. The patients achieving objective responses had significantly lower levels of 3-HAA than those who did not (*p* = 0.045). Receiver operating characteristic analyses determined that the cutoff value of 3-HAA for objective response was 35.4 pmol/mL (sensitivity: 87.5% and specificity: 83.3%). The patients with 3-HAA < 35.4 pmol/mL had significantly longer median progression-free survival (7.0 months) than those without (1.6 months, *p* = 0.022).

**Conclusions:**

Tryptophan metabolites may have a potential for predicting the efficacy of ICIs.

**Registration number:**

University Hospital Medical Information Network Clinical Trial Registry 000026140.

**Electronic supplementary material:**

The online version of this article (10.1007/s12094-020-02421-8) contains supplementary material, which is available to authorized users.

## Introduction

Immune checkpoint inhibitors (ICIs) is increasingly used for a wide variety of cancers [1]. However, ICIs are not effective for all patients. Increased PD-L1 expression in tumor tissue, high tumor mutational burden, or abundant tumor-infiltrating T cells tend to respond better to ICIs; however, these biomarkers were suboptimal to predict responses to ICIs [2]. Thus, developing new biomarkers that can accurately select patients who will benefit from ICIs is strongly desired.

Tryptophan is an essential amino acid, and its metabolites are known to be bioactive compounds that play important roles in several chronic diseases and cancers [3, 4]. Tryptophan metabolites are broadly divided into three major pathways: the serotonin, indole-3-acetic acid, and kynurenine pathways. The majority of free tryptophan is catalyzed by indoleamine-2,3-dioxygenase (IDO) into kynurenine, which is the first and rate-limiting step of the catabolic tryptophan–kynurenine pathway (Supplementary Figure). The tryptophan–kynurenine pathway plays a key role in cancer immunity [5, 6]. Decreased tryptophan induces anergy of effector T cells, and increased kynurenine leads to regulatory T cell activation, which results in the suppression of anti-tumor immune responses, causing cancer development and progression [5]. Additionally, the tryptophan–kynurenine pathway has been reported to be upregulated is a variety of cancers [7]. Moreover, inhibition of this pathway by IDO inhibitors has been intensely investigated as a potential therapeutic target [5].

However, IDO is only the first step of the tryptophan–kynurenine pathway. There are several downstream pathways and metabolites of IDO. Additionally, tryptophan has several alternative pathways other than the tryptophan–kynurenine pathway. It had been extremely difficult to measure the wide variety of tryptophan metabolites simultaneously; therefore, the roles of these diverse metabolites in cancer are largely unknown. Recent advances in mass spectrometric techniques have enabled the comprehensive high-throughput analysis of multiple tryptophan metabolites with accurate quantitative performance [4, 8]. Given the immunoregulatory roles of tryptophan and its metabolites, it was hypothesized that they are associated with the efficacy of ICIs. Using advanced mass spectrometric techniques, this study comprehensively assessed plasma levels of multiple tryptophan metabolites before ICI therapy and elucidated an association between their levels and the efficacy of ICIs in patients with advanced non-small cell lung cancer (NSCLC).

## Materials and methods

### Study design

This is a prospective observational study conducted in accordance with the ethical standards described in the Declaration of Helsinki. The study protocol was approved by the Institutional Review Board of Hamamatsu University School of Medicine (No. 19-083). Each patient provided written informed consent. The study was registered with the University Hospital Medical Information Network Clinical Trial Registry (000,026,140).

### Patient eligibility

Patients with NSCLC who were scheduled for ICI therapy were included. Other inclusion criteria were having an Eastern Cooperative Oncology Group (ECOG) performance status of 0–2 and inoperable stage IIIB or IV or recurrent disease. Patients who had a prior history of immune checkpoint therapy and uncontrolled complications were excluded. Patients who had prior history of chemotherapy were allowed in this study, but those who were scheduled to receive chemotherapy (with or without ICIs) during the study period were excluded. As the control, blood samples from 10 healthy volunteers (all male, median age: 35 years, range: 27–42 years) were also assessed.

### Treatment and evaluation schedule

Blood samples were collected before ICI therapy. ICI therapy was selected by the treating physician (nivolumab or pembrolizumab). Chest computed tomography was performed before and 4 and 8 weeks after ICI therapy and then repeated every 8 weeks until cessation of anti-PD-1 therapy. Radiological response was evaluated according to RECIST version 1.1.

### Measurements of tryptophan metabolites

The precise methods used to measure tryptophan metabolites are described elsewhere [4, 8]. Briefly, plasma levels of tryptophan, kynurenine, serotonin, 5-hydroxyindoleacetic acid, indole-3-acetic acid, anthranilic acid, kynurenic acid, quinaldic acid, 3-indolebutyric acid, 3-hydroykynurenine, 3-hydroxyanthranilic acid (3-HAA), xanthurenic acid, and quinolinic acid were comprehensively measured using liquid chromatography with tandem mass spectrometry (LC–MS/MS; LCMS-8060^®^, Shimadzu, Japan).

### Statistical analyses

The Wilcoxon rank-sum test was used to compare continuous variables. Age- and sex-adjusted logistic regression analysis was performed for comparison between the patients with NSCLC and control subject. Logistic regression and Cox proportional hazard analyses were performed for predictive factors of objective response and progression-free survival, respectively. Kaplan–Meier survival curves and log-rank tests were used to analyze progression-free survival (PFS). The cutoff values of tryptophan metabolites for objective response were determined by receiver operating characteristic (ROC) analysis using Youden’s index. A *p* value < 0.05 (two-sided) was considered significant. All values were analyzed using JMP v13.0.0 (SAS Institute Japan, Tokyo, Japan).

## Results

### Patient characteristics

From September 2016 to December 2018, 19 patients were enrolled in this study. The patient characteristics are shown in (Table 1). Thirteen patients (68.5%) had positive PD-L1 expression with tumor proportion scores (TPS) of ≥ 1% and nine (47.4%) had TPS of ≥ 50%. Twelve (63.2%) and seven (36.8%) patients received nivolumab (as the second line or later therapy) and pembrolizumab (as the first line therapy), respectively. Ten patients (52.7%) achieved objective response to ICIs. The median PFS was 7.0 months [95% confident interval (CI): 1.6 months – not estimated], and the median OS was 12.4 months (95% CI: 7.9–19.5 months).

**Table 1:** Patient characteristics

	*N* = 19
Age	69 (41 − 81)
Sex, male	16 (84.2)
Smoking status, never-/ever-smoker	4 (21.1)/15 (78.9)
ECOG-PS, 0/1/	9 (47.4)/10 (52.6)
Stage, IIIb / IV	1 (5.3)/18 (94.7)
Pathology, adeno / squamous / others	14 (73.7)/3 (15.8)/2 (10.5)
PD-L1 expression, unknown or < 1% / ≥ 1 − < 50% / ≥ 50%	6 (31.5) 4 (21.1)/9 (47.4)
*EGFR* mutation, wild type / del 19	18 (94.7)/1 (5.3)
*ALK* fusion gene, none / unknown	17 (89.5)/2 (10.5)
Treatment line, 1st / ≥ 2nd	7 (36.8)/12 (63.2)
Treatment, nivolumab / pembrolizumab	12 (63.2)/7 (36.8)
Best response to anti-PD-1 therapy PD/SD/PR/CR	(21.1) 5 (26.3)/6 (31.6)/4 (21.1)

Data are expressed as median (range) or number (%)

*ALK* anaplastic lymphoma kinase; *CR* complete response; *ECOG-PS* Eastern Cooperative Oncology Group performance status; *EGFR* epidermal growth factor receptor; *PD-1* programmed cell death-1; *PD-L1* programmed cell death-ligand 1; *PD* progressive disease; *PR* partial response; *SD* stable disease

### Association between tryptophan metabolites and the efficacy of ICI therapy

**Fig. 1:**
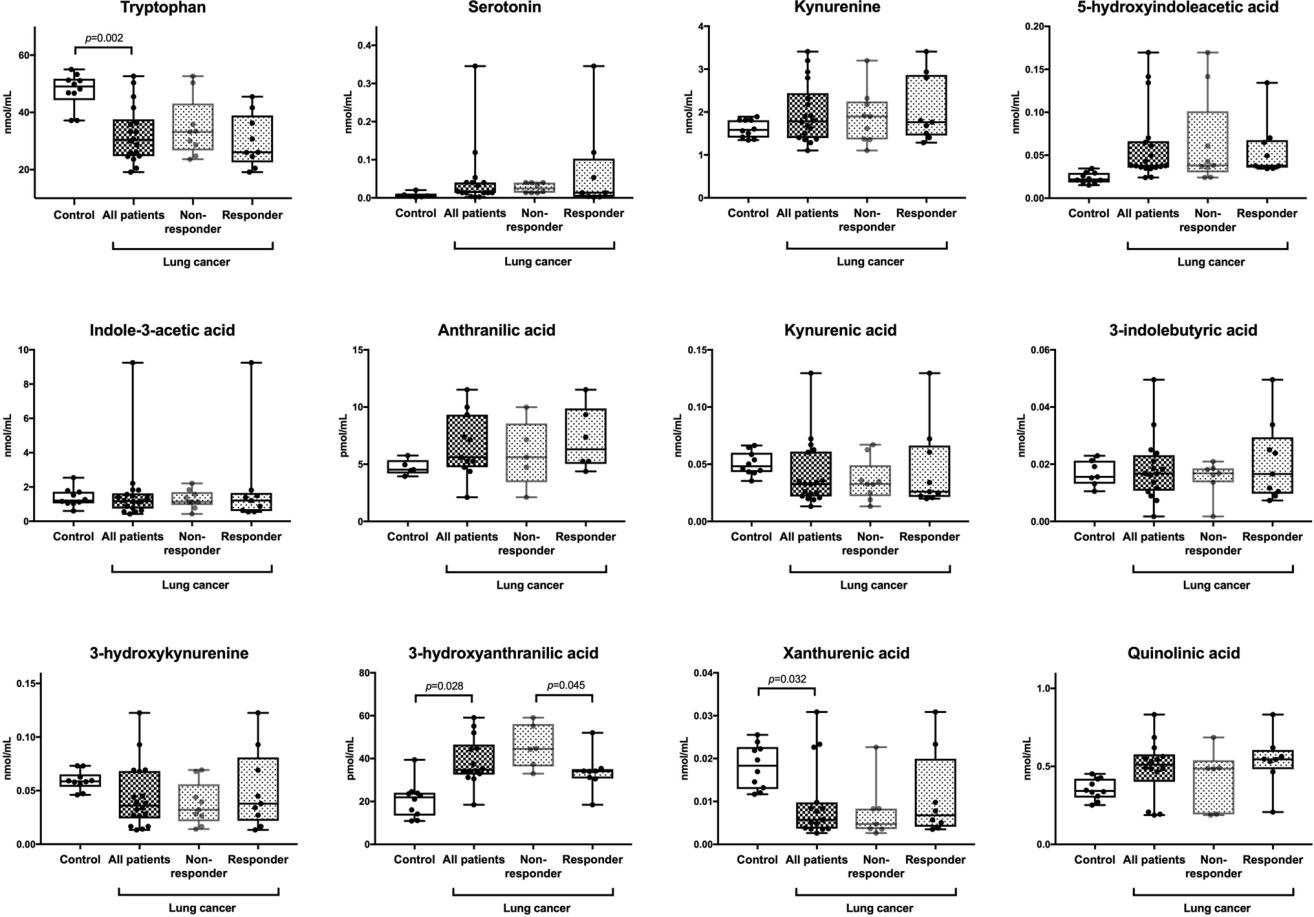
Plasma tryptophan metabolites in patients with non-small cell lung cancer and healthy controls. Responders and non-responders were defined as patients who achieved objective response and those who did not, respectively. Horizontal lines, boxes, and error bars represent the median, the 25th and 75th percentiles, and the minimum and the maximum, respectively

**Fig. 2:**
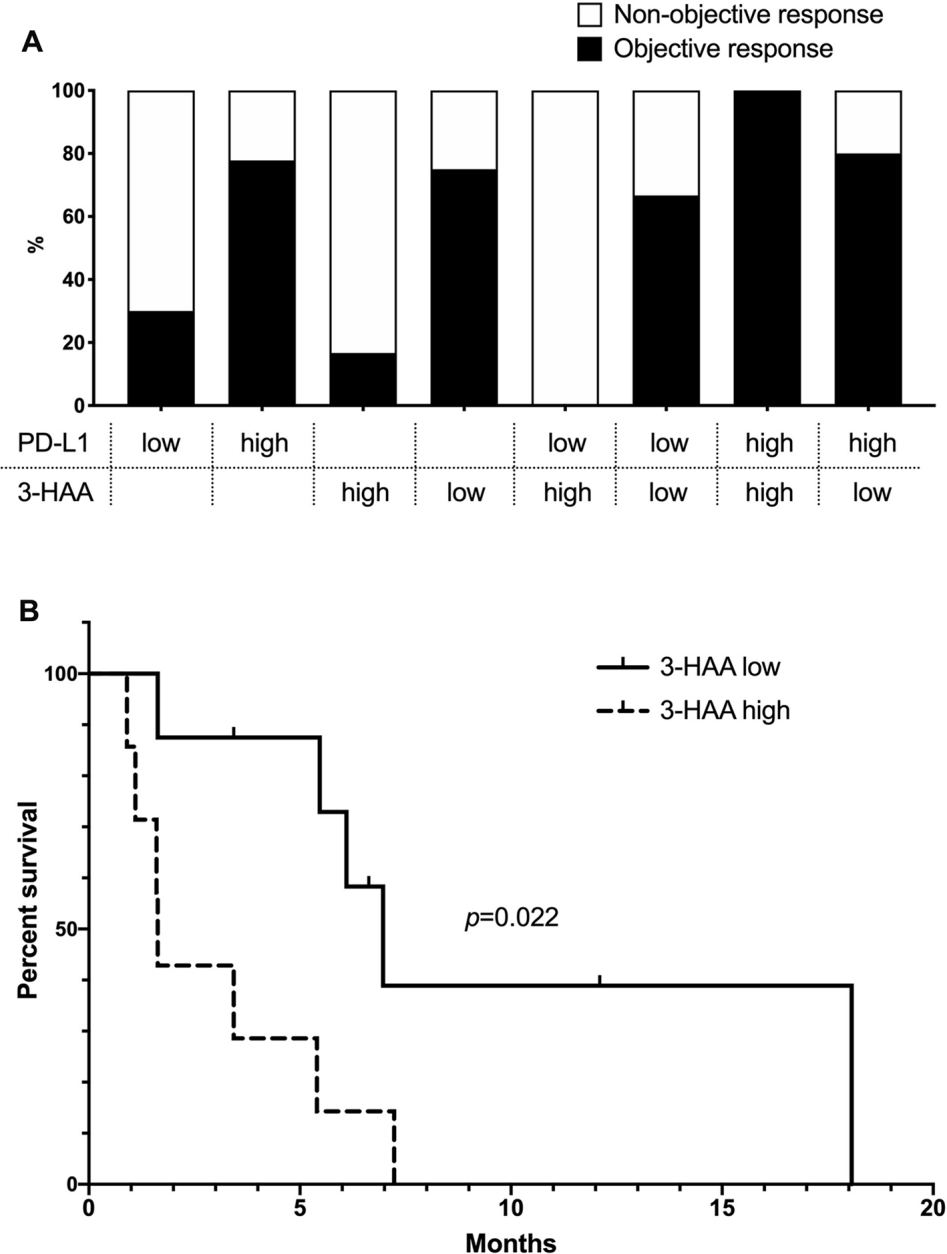
Plasma 3-hydroxyanthranilic acid levels and the efficacy of immune check point inhibitors. (**a**) Objective response rate according to the status of programmed death-ligand 1 (PD-L1) and/or 3-hydroxyanthranilic acid (3-HAA). (**b**) Progression-free survival in the patients with high and low 3-HAA levels. High PD-L1 expression and low 3-HAA levels were defined as a tumor proportion score of ≥ 50%, and plasma level of < 35.4 pmol/mL, respectively

Compared with the control subjects, the patients with NSCLC demonstrated significantly lower levels of tryptophan (*p* = 0.002) and xanthurenic acid (*p* = 0.032), and significantly higher levels of 3-HAA (*p* = 0.028) (Fig. 1). The patients who achieved objective responses demonstrated significantly lower levels of 3-HAA than those who did not (*p* = 0.045). The other tryptophan metabolites did not have a significant correlation with responses to ICIs. In ROC analysis, the cutoff value of 3-HAA for objective response was 35.4 pmol/mL (sensitivity: 87.5%, 95%CI: 42.1–99.6%; specificity: 83.3%, 95%CI: 29.0–96.3%; and AUC: 0.83). High PD-L1 expression of TPS ≥ 50% had a sensitivity of 70.0% (95% CI: 34.5–93.3%) and a specificity of 77.8% (95%CI: 40.0–97.2%) for predicting objective responses. Among the seven patients who demonstrated objective responses, two (28.6%) did not have high PD-L1 expression of TPS ≥ 50% but had 3-HAA < 35.4 pmol/mL (Fig. 2a). When used in combination, patients with either PD-L1 TPS ≥ 50% or 3-HAA < 35.4 pmol/mL demonstrated a sensitivity of 100% (95% CI: 47.3–100%) and a specificity of 71.4% (95%CI: 29.0–96.3%). The patients with 3-HAA < 35.4 pmol/mL had significantly longer median PFS (7.0 months) than those with 3-HAA ≥ 35.4 pmol/mL (1.6 months, *p* = 0.022) (Fig. 2b). In multivariate analyses, 3-HAA < 35.4 pmol/mL was a significant predictive factor for progression-free survival (*p* = 0.013) but was not for objective response (*p* = 0.144) (Supplementary Table 1, 2).

## Discussion

This is the first study to comprehensively assess multiple tryptophan metabolites in patients with NSCLC receiving ICI therapy. Interestingly, several plasma tryptophan metabolites were altered in patients with NSCLC compared with control subjects. Furthermore, the patients who achieved objective responses demonstrated a significantly lower level of 3-HAA than those who did not. Quantitation of 3-HAA had a high accuracy for the prediction of ICI efficacy, which was further increased when combined with PD-L1 expression. Additionally, the patients with low 3-HAA (< 35.4 pmol/mL) had significantly longer PFS than those with high 3-HAA. Taken together, these observations suggest that an assessment of tryptophan metabolites is helpful for predicting the efficacy of ICI therapy in patients with NSCLC.

3-HAA is a downstream metabolite of the kynurenine pathway (Supplementary Figure). Kynurenine hydroxylase catalyzes kynurenine into 3-hydroxykynurenine, and then kynureninase B catalyzes 3-hydroxykynurenine into 3-HAA [3, 8]. Although its precise role in cancer immunity is unclear, 3-HAA is known to have anti-inflammatory activity [9–12]. Gargaro et al. reported that 3-HAA induced regulatory T cells via the production of transforming growth factor β, decreasing the number of effector T cells [12]. Considering the immunosuppressive activity of 3-HAA, it is reasonable that a lower 3-HAA levels will show better responses to ICIs than those without. PD-L1 expression in tumor tissues is a key factor in the therapeutic efficacy of ICIs; however, the host immune response to cancer cells is also necessary [13]. In this study, the combination of PD-L1 and 3-HAA had better predictive accuracy than either of the two alone. Collectively, the assessment of plasma tryptophan metabolites using advanced LC–MS/MS is a non-invasive and repeatable method that has a potential clinical utility in predicting the efficacy of ICIs.

Tryptophan metabolism is not regulated by cancer immunity alone, but by several health conditions and chronic diseases [3, 4, 8]. However, in addition to 3-HAA and kynurenine, several other tryptophan metabolites are known to have immunosuppressive functions [14–16]. Thus, tryptophan metabolites may have potential not only to evaluate anti-cancer immunity but also to predict the therapeutic efficacy of ICIs. Furthermore, the tryptophan synthesis pathway could be a novel target for immune therapy. Future studies will reveal the precise mechanisms that regulate tryptophan metabolism and its immunoregulatory roles in cancer patients.

This study had three main limitations. First, the actual roles of tryptophan metabolites in cancer immunity are still unknown. Associations between tryptophan metabolites and host immune status (e.g., effecter T cells, regulatory T cells, or dendritic cells) should be investigated in the future. Second, there was no established cutoff value of 3-HAA, and the value determined by ROC analysis was exploratory. Third, this study evaluated single-agent therapy with anti-PD-1 antibodies in a limited number of patients with NSCLC. Recently, novel ICI therapies have emerged for cancer therapy, such as anti-PD-L1 antibody, combination therapy with two different ICIs, and combination of cytotoxic agents and ICIs [17–19]. Further studies are needed in a larger number of patients with different types of immunotherapies and cancers other than NSCLC to validate the utility measuring tryptophan metabolites.

## Conclusions

Patients with NSCLC had different plasma levels of several tryptophan metabolites compared with healthy controls. Among them, lower 3-HAA levels were associated with better objective responses and longer PFS; thus, 3-HAA may be helpful for predicting the efficacy of ICIs.

## Electronic supplementary material

Below is the link to the electronic supplementary material.

Supplementary file1 (DOCX 1020 kb)

Supplementary file2 (DOCX 38 kb)
