# Supplementary figures and images for: Comprehensive assessment of multiple tryptophan metabolites as potential biomarkers for immune checkpoint inhibitors in patients with non-small cell lung cancer
Source: Clin Transl Oncol. 2020 Jun 12;23(2):418–23. doi: 10.1007/s12094-020-02421-8 (PMC7854397; doi:10.1007/s12094-020-02421-8)

**Supplementary Figure. A schematic diagram of tryptophan metabolites**

**
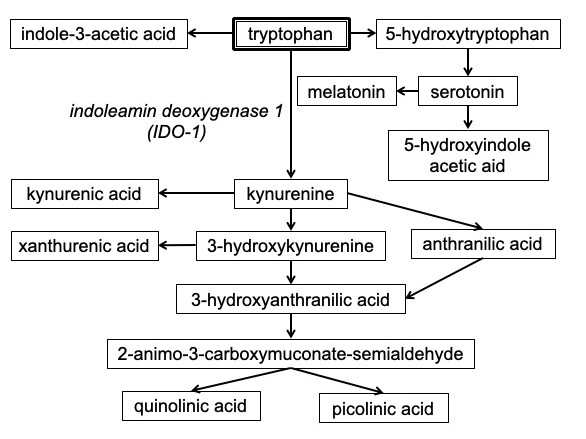
**

Supplement: Supplementary file 1 — Supplementary file1 (DOCX 1020 kb) [file 12094_2020_2421_MOESM1_ESM.docx]
