# Supplementary material for: Comprehensive assessment of multiple tryptophan metabolites as potential biomarkers for immune checkpoint inhibitors in patients with non-small cell lung cancer
Source: Clin Transl Oncol. 2020 Jun 12;23(2):418–23. doi: 10.1007/s12094-020-02421-8 (PMC7854397; doi:10.1007/s12094-020-02421-8)
Supplement: Supplementary file 2 — Supplementary file2 (DOCX 38 kb) [file 12094_2020_2421_MOESM2_ESM.docx]

**Supplementary Table 1. Logistic regression analyses of objective response**

|  | Univariate | | Multivariate | |
| --- | --- | --- | --- | --- |
| Variables | Odds ratio | *p*-value | Odds ratio | *p*-value |
| Age, ≤65 years | 0.88 (0.10 − 7.95) | 0.906 |  |  |
| Sex, male | 6.55×10^7^ (N.E.) | 0.996 |  |  |
| Smoking, ever-smoker | 8.12×10^7^ (N.E.) | 0.995 |  |  |
| ECOG-PS, 0 (vs. ≥1) | 0.80 (0.13 − 4.87) | 0.809 |  |  |
| Pathology, squamous cell  (vs. non-squamous) | 2.00 (0.15 − 26.73) | 0.600 |  |  |
| Stage, IIIb (vs. IV) | 8.18×10^6^ (N.E.) | 0.996 |  |  |
| PD-L1 expression |  |  |  |  |
| TPS≥1% | 3.20 (0.42 – 24.42) | 0.262 |  |  |
| TPS≥50% | 8.17 (1.03 – 64.94) | 0.047 | 9.00 (0.47 – 171.64) | 0.144 |
| Treatment line, 1st (vs. ≥2nd) | 3.50 (0.47 – 25.90) | 0.212 |  |  |
| 3-HAA, <35.4 pmol/mL (vs. ≥35.4 pmol/mL) | 15.00 (1.03 – 218.30) | 0.048 | 9.00 (0.47 – 171.64) | 0.144 |

Data are expressed as odds ratio (95% confident interval).

ECOG-PS, Eastern Cooperative Oncology Group performance status; N.E.; not estimated; PD-L1, programmed cell death-ligand 1; TPS, tissue proportion score; 3-HAA, 3-hydroxyanthranilic acid.

**Supplementary Table 2. Cox proportional hazard analyses of progression-free survival**

|  | Univariate | | Multivariate | |
| --- | --- | --- | --- | --- |
| Variables | Hazard ratio | *p*-value | Hazard ratio | *p*-value |
| Age, ≤65 years | 0.91 (0.20 − 3.15) | 0.894 |  |  |
| Sex, male | 0.91 (0.23 − 5.96) | 0.902 |  |  |
| Smoking, ever-smoker | 0.64 (0.18 − 2.94) | 0.527 |  |  |
| ECOG-PS, 0 (vs. ≥1) | 0.71 (0.22 – 2.35) | 0.571 |  |  |
| Pathology, squamous cell  (vs. non-squamous) | 0.29 (0.02 – 1.54) | 0.167 |  |  |
| Stage, IIIb (vs. IV) | 1.97 (0.10 − 11.12) | 0.562 |  |  |
| PD-L1 expression | 0.73 (0.11 − 2.95) | 0.682 |  |  |
| TPS≥1% | 0.86 (0.27 – 3.26) | 0.809 |  |  |
| TPS≥50% | 0.35 (0.09 – 1.12) | 0.077 |  |  |
| Treatment line, 1st (vs. ≥2nd) | 0.24 (0.04 – 0.94) | 0.040 | 0.14 (0.01 – 0.74) | 0.018 |
| 3-HAA, <35.4 pmol/mL (vs. ≥35.4 pmol/mL) | 0.25 (0.06 – 0.89) | 0.034 | 0.15 (0.02 – 0.68) | 0.013 |

Data are expressed as hazard ratio (95% confident interval).

ECOG-PS, Eastern Cooperative Oncology Group performance status; PD-L1, programmed cell death-ligand 1; TPS, tissue proportion score; 3-HAA, 3-hydroxyanthranilic acid.
